# Conservative treatment of patients with bladder genital tract fistula: Three case reports

**DOI:** 10.1097/MD.0000000000021430

**Published:** 2020-07-31

**Authors:** Zhiwei He, Lifeng Cui, Jia Wang, Fengyan Gong, Guifeng Jia

**Affiliations:** Department of Gynecology and Obsterics, The First Hospital of Jilin University, Changchun, Jilin, China.

**Keywords:** bladder genital tract fistula, case report, conservative treatment, gynecology

## Abstract

**Introduction:**

Most of the patients with bladder genital tract fistula recover with surgical treatment. In the present study, we aimed to assess conservative treatment strategies for bladder genital tract fistula.

**Patient concerns:**

We reviewed 3 cases with bladder genital tract fistula who underwent treatment at our hospital from January to June 2017. Patient 1 underwent cesarean delivery, Patient 2 underwent total abdominal hysterectomy bilateral salpingo-oophorectomy (TAHBSO) and pelvic lymphadenectomy, and Patient 3 underwent extensive TAHBSO and pelvic lymphadenectomy. All 3 patients exhibited involuntary vaginal fluid outflow (average duration, 12.7 days; range, 7–21 days).

**Diagnosis:**

Patient 1 was diagnosed as vesicouterine fistula by cystosonography and Patient 2, Patient 3 was diagnosed as vesicovaginal fistula by cystoscopy.

**Interventions:**

All 3 patients underwent indwelling urinary catheterization.

**Outcomes:**

No vaginal fluid outflow could be observed after treatment of all 3 patients.

**Conclusion:**

Indwelling urinary catheterization should be administered for suitable patients as conservative treatment. If vesicouterine fistulas that are simple and have a diameter of <0.5 cm can be treated conservatively. If the condition does not resolve after 2 months, surgery should be considered.

## 1 Introduction

Female urogenital fistula, also termed as urinary fistula, refers to abnormal tracts that develop at sites adjacent to the vagina, bladder, ureters, or urethra. Involuntary excretion of urine from the vagina is a major symptom of female urogenital fistulas.^[1]^ Urinary fistulas may develop at any site adjacent to the urinary tract, and vesicovaginal fistula is the most common type.^[2]^ Most female patients with vesicovaginal fistulas can recover well with surgical treatment. However, such treatment is associated with great financial burden to the families, and often requires secondary surgery. In certain patients, the repair surgery is performed 3 to 6 months after the first surgery, and is associated with great physical and psychological trauma. In the present report, we discuss conservative treatment strategies of 3 cases with bladder genital tract fistula.

## 2 Case report

### 2.1 Case 1

A 32-year-old patient (Patient 1) was diagnosed with abnormal healing of an abdominal incision following cesarean delivery. Seven days after surgery, she developed involuntary urinary fluid outflow, and underwent further treatment after 12 days. She underwent cystosonography, and contrast could be observed at the isthmus area of the anterior uterine wall. Her sinus was 4 mm in length and 5 mm in width. Thereafter, contrast was observed through the uterine cavity and vagina. The patient was diagnosed as vesicouterine fistula following cystosonography, and as abnormal healing of the abdominal incision. Following treatment with antibiotics, incision dressing change, and indwelling urinary catheterization, she underwent cystoscopy and bilateral ureteral stent implantation. During surgery, thorough examination of the bladder indicated a 0.1 to 0.2-cm cleft at the upper portion of the posterior wall of the urinary bladder; hyperemia and edema were also observed at this site. Hence, the cleft was determined as a vesicouterine fistula. After surgery, she underwent treatment with antibiotics, bladder irrigation, and indwelling urinary catheterization. Once the infection was under control, she underwent laparotomy. The patient was scheduled to undergo excision and repair of the sinus tract between the bladder and uterine anterior wall; however, during the surgery, the bladder uterine sinus was not visible. As we could only view the sinus between the abdominal incision and uterine anterior wall, she underwent excision of the sinus between the abdominal incision and uterine anterior wall, along with abdominal incision sutures. Thereafter, she underwent treatment with antibiotics, bladder irrigation, and indwelling urinary catheterization. She underwent cystoscopy 51 days after indwelling urinary catheterization. As no clear fistula could be observed, we removed the bilateral ureteral stent and discontinued the indwelling urinary catheterization. No vaginal fluid outflow or relapse was observed after 6 months based on the patient's follow-up visits.

### 2.2 Case 2

A 46-year-old patient (Patient 2) with endometrial cancer underwent total abdominal hysterectomy bilateral salpingo-oophorectomy (TAHBAO) and pelvic lymphadenectomy. Twenty-one days after surgery (11 days after chemotherapy hydration), she exhibited involuntary urinary fluid outflow. Six days later, she underwent cystoscopy, and a 3 × 4 mm-sized fistula, hyperemia, and edema were observed at the posterior wall of the urinary bladder. She was diagnosed as vesicovaginal fistula, and underwent indwelling urinary catheterization. She did not show any obvious vaginal fluid outflow 60 days after indwelling urinary catheterization, and no relapse was observed after indwelling urinary catheterization was discontinued.

### 2.3 Case 3

A 49-year-old patient (Patient 3) with cervical cancer underwent bilateral ureteral stent implantation under cystoscopy, extensive TAHBAO, and pelvic lymphadenectomy. Ten days after surgery, she exhibited involuntary urinary fluid outflow. The vaginal fluid was tested, and the results are presented in Table 1. Following cystoscopy, she was diagnosed as vesicovaginal fistula, although the location of the fistula was unclear. After surgery, she underwent indwelling urinary catheterization. Moreover, she received bladder irrigation. She underwent cystosonography 28 days after indwelling urinary catheterization. As no obvious sinus could be observed, we removed the bilateral ureteral stent and terminated the indwelling urinary catheterization. No vaginal fluid outflow could be observed after treatment. She was continuously administered chemotherapy, but no relapse of vaginal fluid outflow was noted after 3 months based on patient's follow-up visits.

**Table 1:**
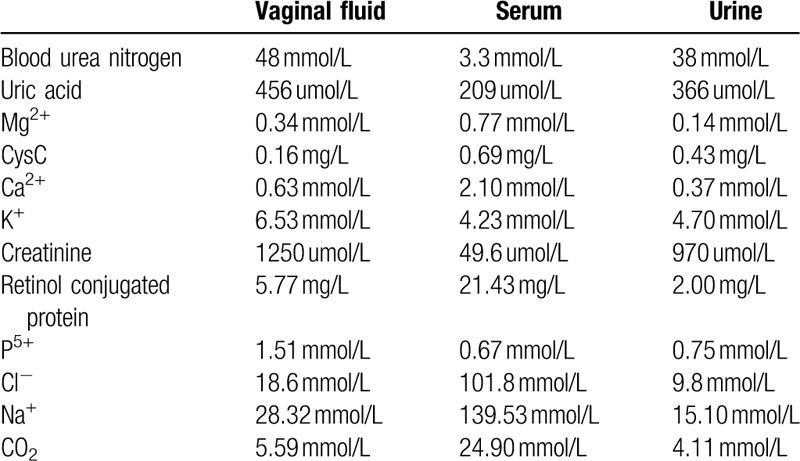
Examination of vaginal fluid using serum and urine biochemical tests in Patient 3.

Detailed information regarding these cases can be observed in Table 2. All the cases were recommended to improve their nutritional intake and drink more water. Patient 1 had fever, but none of the patients had any stomach pain.

**Table 2:**
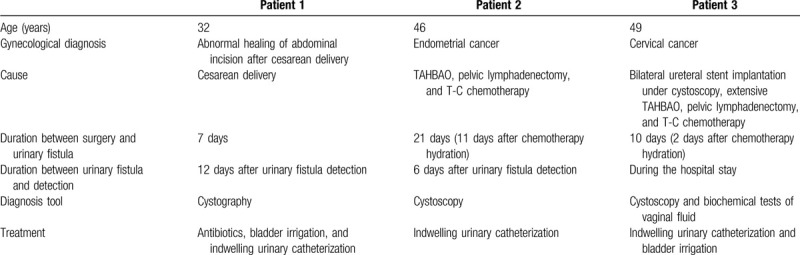
Detailed information about the 3 cases.

## 3 Discussion

The bladder is a hollow organ located adjacent to the uterus, rectum, and other important organs. Urinary fistulas are abnormal tracts that develop at sites between the genital tract and bladder. According to lesion severity, urinary fistulas can be classified as simple urinary fistulas, complex urinary fistulas, and extra-complex urinary fistulas. Simple urinary fistulas may involve vesicouterine fistulas with a diameter of <3 cm or urethrovaginal fistulas with a diameter of <1 cm. Complex urinary fistulas may involve vesicouterine fistulas with a diameter of >3 cm or urethrovaginal fistulas with a diameter of >1 cm (or with a distance of <0.5 cm between the verge of the fistula and ureteral opening). Other rare types of urinary fistulas are classified as extra-complex urinary fistulas.^[1]^ Vesicouterine fistulas are the most common types of urinary fistula, and are primarily located adjacent to the bladder neck, trigone of the bladder, and lower portion of the bladder.^[3]^

The common etiologies for vesicouterine fistula include surgical injury, obstetrical injury, radioactive damage, and advanced malignant tumor violation.^[4,5]^ With the continuous recent improvements in obstetrics, cases of vesicouterine fistula caused by obstetrical surgical have been decreasing. However, injury during gynecological surgery has become the main reason for vesicouterine fistula.^[6]^ Doganov^[7]^ et al found that urinary fistulas are mainly caused after total hysterectomy and pelvic lymphadenectomy. Patients 1 in the present study possibly developed the bladder injury during a cesarean section. However, it is also possible that avascular necrosis of the bladder may have caused the bladder injury. In the other 2 cases, we believe that urinary fistula developed due to pelvic adhesions, given the large scope of the prior gynecological surgery in those cases. The anatomical close location of the bladder and uterus were also related.

In addition to the clinical manifestation of involuntary urine outflow from the vagina, the diagnosis of urinary fistula requires further examinations, including routine urine tests, biochemical tests, cystoscopy, colposcopy, intravenous urography, and retrograde urography. Moreover, the patient needs to undergo the indigo carmine test to clarify the diagnosis of urinary fistula, and determine the location.^[1]^ Among the present cases, Patient 1 underwent cystosonography before treatment, while Patient 3 underwent cystosonography after treatment, and the findings were useful for the clinical diagnosis, treatment decision and prognosis evaluation.

Following the diagnosis of vesicouterine fistulas, most patients undergo surgical treatment.^[8]^ Surgical repair is the routine treatment method for urinary fistula and has exhibited positive curative effects. The surgery is usually performed 3 to 6 months after the initial surgery, as this ensures sufficient time for recovery of the inflammation and edema around the sinus. At this time, the patients usually develop urine immersion eczema, and the involuntary urine outflow is associated with impairment of the patients’ daily routine and working activity. If conservative treatment can achieve clinical recovery in these cases, the secondary surgery can be avoided, and the discomfort caused by involuntary urine outflow can be resolved at an early stage. Research has shown that it is possible to achieve recovery through treatment with a smooth drainage catheter, stronger antibiotics, anticholinergic and antispasmodic drug use, and other conservative treatment in patients with a small vesicouterine fistula.^[8]^ Shi Qigang^[9]^ et al recommended that certain vesicouterine fistula patients with a fistula diameter of ≤0.5 cm can undergo conservative treatment with indwelling urinary catheterization and antibiotics. Zhou Rong^[10]^ et al reported 2 cases of vesicouterine fistula with a fistula diameter of 0.5 cm who recovered 2 months after conservative treatment. The 3 cases in the present study were believed to be caused by surgery. Based on the patients’ condition, fistulas caused by surgery are easier to recover, as compared to those resulting from avascular necrosis. Patient 1 underwent cystosonography, and contrast could be observed at the isthmus area of the anterior uterine wall. Her sinus was 4 mm in length and 5 mm in width. Patient 2 and Patient 3 underwent cystoscopy and exhibited fistulas with a size of 4 × 5 mm and 3 × 4 mm; the diameters in those cases were both <0.5 cm. Patient 1 underwent cystoscopy, but no obvious fistula was observed; however, edema and congestion could be noted. It is considered that granulation tissue has already grown. All the fistulas in the present report could be classified as simple urinary fistula. Moreover, the 3 patients underwent indwelling urinary catheterization to ensure that the bladder was empty and thus decrease urinary stimulation to the fistula. Moreover, the patients were recommended to improve their nutritional intake and drink more water. Furthermore, the patients received bladder irrigation to reduce the incidence of bladder inflammation. Following 2 months of involuntary urine outflow, the 3 patients exhibited no obvious symptoms of urinary outflow and exhibited clinical recovery. Among these patients, Patient 3 did not show any obvious sinus on cystosonography. No relapse was observed after 3 months based on the patient's follow-up. The other 2 patients did not undergo cystosonography after treatment, and showed no relapse.

We believe that vesicouterine fistulas that are simple and have a diameter of <0.5 cm can be treated conservatively. If the condition does not resolve after 2 months, surgery should be considered. However, reports of such instances are rare. In the present report, we describe only 3 cases, which may not be sufficient to prove that conservative treatment is effective for urinary fistulas. Hence, a larger study with a longer follow-up period would be useful.

## Author contributions

**Conceptualization:** Fengyan Gong.

**Data curation:** Fengyan Gong.

**Project administration:** Jia Wang.

**Supervision:** Guifeng Jia.

**Writing – original draft:** Zhiwei He.

**Writing – review & editing:** Lifeng Cui.
